# The Hemodynamic Mechanism of FFR-Guided Coronary Artery Bypass Grafting

**DOI:** 10.3389/fphys.2021.503687

**Published:** 2021-02-05

**Authors:** Bao Li, Boyan Mao, Yue Feng, Jincheng Liu, Zhou Zhao, Mengyao Duan, Youjun Liu

**Affiliations:** ^1^College of Life Science and Bio-Engineering, Beijing University of Technology, Beijing, China; ^2^The School of Life Sciences, Beijing University of Chinese Medicine, Beijing, China; ^3^Department of Medical Equipment, Peking University First Hospital, Beijing, China; ^4^Cardiac Surgery Department, PeKing University People’s Hospital, Beijing, China

**Keywords:** fractional flow reserve, coronary artery bypass grafting, hemodynamics, multiscale model, lumped parameter model

## Abstract

Clinically, fractional flow reserve (FFR)-guided coronary artery bypass grafting (CABG) is more effective than CABG guided by coronary angiography alone. However, no scholars have explained the mechanism from the perspective of hemodynamics. Two patients were clinically selected; their angiography showed 70% coronary stenosis, and the FFRs were 0.7 (patient 1) and 0.95 (patient 2). The FFR non-invasive computational model of the two patients was constructed by a 0–3D coupled multiscaled model, in order to verify that the model can accurately calculate the FFR results. Virtual bypass surgery was performed on these two stenoses, and a CABG multiscaled model was constructed. The flow rate of the graft and the stenosis coronary artery, as well as the wall shear stress (WSS) and the oscillatory shear index (OSI) in the graft were calculated. The non-invasive calculation results of FFR are 0.67 and 0.91, which are close to the clinical results, which proves that our model is accurate. According to the CABG model, the flow ratios of the stenosis coronary artery to the graft of patient 1 and patient 2 were 0.12 and 0.42, respectively. The time-average wall shear stress (TAWSS) results of patient 1 and patient 2 grafts were 2.09 and 2.16 Pa, respectively, and WSS showed uniform distribution on the grafts. The OSI results of patients 1 and 2 grafts were 0.0375 and 0.1264, respectively, and a significantly high OSI region appeared at the anastomosis of patient 2. The FFR value of the stenosis should be considered when performing bypass surgery. When the stenosis of high FFR values is grafted, a high OSI region is created at the graft, especially at the anastomosis. In the long term, this can cause anastomotic blockage and graft failure.

## Introduction

Coronary artery bypass grafting (CABG) is a common surgical procedure for the treatment of myocardial ischemia (Beck, 1935; Vineberg, 1948). The surgeon uses an artery or vein of the patient to anastomose from the aorta to the distal end of the stenosis, so that blood flow can directly supply the distal myocardium through the graft, thereby achieving the purpose of treating myocardial ischemia. The main problem with CABG is the failure of the graft. One of the main reasons for graft failure, aside from technical causes, is that it anastomoses a less severe stenosis (Pellicano et al., 2010). Studies have shown that when the graft is anastomosed to a moderate stenosis, the damage rate is very high (Hanet et al., 1991; Sabik et al., 2003; Berger, 2004).

At present, it is mainly through imaging data, such as coronary angiography, that determines whether the stenosis is serious. However, some researchers used the fluorescent particle method to observe the graft and target coronary artery after CABG, finding that anatomical stenosis does not necessarily result in functional ischemia. A false estimate of the degree of ischemia affects the blood flow of the graft (Ferguson et al., 2013). Therefore, we need to functionally evaluate a stenosis. In this regard, fractional flow reserve (FFR) has been shown to be an effective complement to coronary angiography, which can determine whether coronary stenosis can actually trigger myocardial ischemia (Pijls et al., 1995, Pijls et al., 1996).

FFR has been widely used by cardiologists to guide percutaneous coronary intervention (PCI). But its use to guide CABG is still a very new area of research. Botman et al. (2007) prospectively studied the vascular permeability of 164 patients who underwent CABG 1 year after surgery and found that FFR-guided surgery had a graft failure rate of about one half of that under angiographic guidance. A study by Toth et al. (2013) found that doctors used FFR to guide surgical decisions, using fewer grafts for patients, and the incidence of angina pectoris in patients with FFR-guided CABG was significantly lower than that of the angiographic group after 3 years. In a follow-up study, they also found that CABG under FFR guidance had a significantly lower incidence of cardiovascular events after 6 years than the angiographic guide group (Stephane et al., 2018). The FFR guidelines play an important role in determining whether an angiogram shows moderate stenosis lesions requiring bypass and whether these lesions can affect the long-term effectiveness of grafting (Casselman et al., 2016).

According to the study, hemodynamics is a key factor affecting the permeability of the graft. The failure of the graft is mainly caused by atherosclerosis and intimal hyperplasia (Whittemore et al., 1981; Butany et al., 1998); poor hemodynamic factors are considered to be the most important factors in their occurrence and development (Bassiouny et al., 1992; Hofer et al., 1996). Therefore, it is clear that FFR guides the hemodynamic mechanism behind CABG, which can help to understand why FFR technology can help improve the permeability of graft and lay the foundation for further development of this technology.

## Materials and Methods

This study is aimed at the moderately sensitive stenosis of FFR; two patients with the same stenosis rate but different FFR values were selected. The 3D model of two patients’ coronary artery and bypass grafting were reconstructed to conduct the simulation, respectively. The hemodynamic parameters in the graft of these two patients were obtained by numerical simulation to correctly analyze the hemodynamic mechanism of FFR-guided CABG.

### Patient Clinical Data

In this study, two patients with moderate stenosis diagnosed by angiography were selected, and they also underwent invasive FFR. The stenosis of the first patient (patient 1) was located on the left anterior descending branch (LAD), and the stenosis of the second patient (patient 2) was located in the right coronary artery (RCA). Both stenosis diagnoses showed a stenosis rate of 70%. However, patient 1 had an FFR detection of 0.7 and patient 2 had a FFR of 0.95.

At the same time, we also obtained preoperative computed tomography angiography (CTA) data, as well as clinical basic information such as gender, age, heart rate, blood pressure, and cardiac output. Patient specific information is shown in Table 1.

**TABLE 1:** Patient clinical data.

	**Gender**	**Age**	**Heart**	**Blood**	**Cardiac**	**Stenosis**	**FFR**
			**rate**	**pressure**	**output**	**rate**	
			**(times/min)**	**(mmHg)**	**(ml/min)**		
Patient 1	Male	56	77	120/70	4,675.6	70%	0.7
Patient 2	Male	53	62	124/74	5,032.3	70%	0.95

### Construction of FFR Non-invasive Computing Model

In order to study the hemodynamic effects of FFR on CABG surgery, we first need to construct a 0–3D coupled multi-scaled model with an FFR non-invasive calculation. The FFR non-invasive calculation method used in this study is based on the research results of Taylor et al. (2013), and its accuracy has been fully confirmed.

**FIGURE 1:**
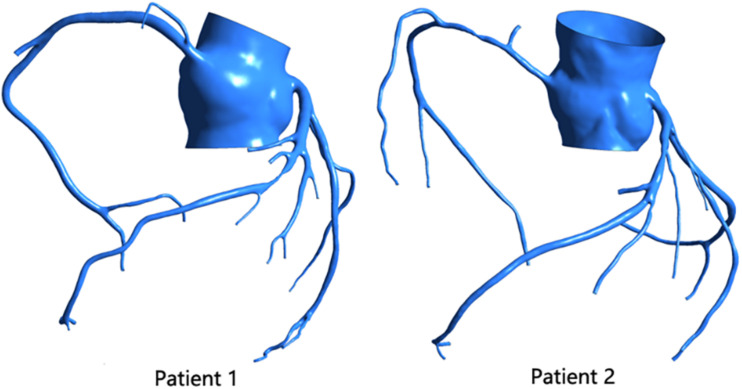
Patient’s coronary 3D model.

Using the patient’s CTA data, we reconstructed a coronary 3D model of two patients, as shown in Figure 1. Mimics 17.0 was used in this study for 3D reconstruction. After the CTA image was imported into the software, the aorta and coronary artery regions were selected by threshold segmentation. The aorta and main coronary arteries could be separated by selecting the appropriate threshold. For small distal coronary arteries, manual adjustment was required. After selecting the desired region, the 3D model of the coronary artery system was reconstructed. When 3D reconstruction of the coronary arteries was completed, the virtual surgical software Freeform was used to smooth the surface of the model, and then the reconstructed 3D model of the patient was obtained.

The model was meshed by the ANSYS ICEM CFD software. The models were meshed by the tetrahedral mesh method. The drawn mesh passed the grid sensitivity analysis. For the 3D model of the coronary artery, we assumed that the vessel wall was rigid, and the material properties of blood were set as adiabatic, isotropic, and incompressible Newtonian fluids. Its hemodynamic viscosity was 0.0035 Pas, and its density was 1,050 kg/m^3^.

**FIGURE 2:**
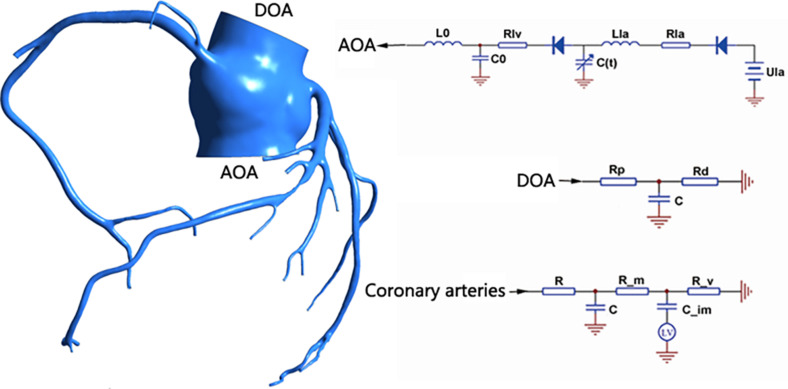
0–3D coupled multi-scale model.

The multi-scale model coupled the 0D model to the 3D model, and the 0D model provided boundary conditions for the 3D model, as shown in Figure 2 (taking patient 1 as an example). The 0D model uses the circuit structure to simulate the vascular network, simplifying the complex three-dimensional blood flow problem into a simple circuit-solving problem. We used resistors to simulate blood flow resistance, capacitors to simulate blood vessel elasticity, and inductors to simulate blood flow inertia.

The 0D model in Figure 2 can be divided into three modules: the heart module, the aortic module, and the coronary module.

For the heart module, the purpose was to provide boundary conditions for the aortic inlet of the 3D model, so our heart model only included the left part of the heart and left out the right part. In the left heart, the blood enters the left ventricle through the left atrium via the mitral valve. Blood is pumped through the aortic valve to the aorta by compression of the left ventricle, which then completes the systemic circulation. The Ula part is a constant voltage source, which represents the pressure of the left atrium. This is because the pressure of the left atrium is small and does not fluctuate significantly with the cardiac cycle, so it can be replaced by a constant voltage source. The two diodes represent the mitral valve and the aortic valve from right to left, respectively, to ensure the single conduction of current. The resistance Rla and inductance Lla, respectively, represent the flow resistance and flow inertia through the mitral valve. Resistance Rlv represents the resistance of the blood flow through the aortic valve. C(t) is a time-varying capacitance that reflects the periodic contraction and relaxation of the left ventricle, and the change of its value is regulated by the relationship between the pressure and volume of the left ventricle. The functional relationship of *C*(*t*) is as follows:

(1) $$C\,\left(t\right)=\frac{1}{E\,\left(t\right)}$$

(2) $$E\,\left(t\right)=\left(E_{m\,a\,x}-E_{m\,i\,n}\right)\cdot E_{n}\,\left(t_{n}\right)+E_{m\,i\,n}$$

(3) $$E_{n}\,\left(t_{n}\right)=1.55\,\left[\frac{{\left(\frac{t_{n}}{0.7}\right)}^{1.9}}{1+{\left(\frac{t_{n}}{0.7}\right)}^{1.9}}\right]\,\left[\frac{1}{1+{\left(\frac{t_{n}}{1.17}\right)}^{21.9}}\right](\mathrm{Stergiopuloset al}.,1996)$$

Among them, $t_{n}=\frac{t}{T_{m\,a\,x}},T_{m\,a\,x}=0.2+0.15\,t_{c},t_{c}$ represents the time of a cardiac cycle.

For the aortic module, the resistance *R_P* represents the arterial blood flow resistance; the resistance Rd represents the sum of the arterial end, the microcirculatory system, and the venous system resistance; and the capacitance *C* represents the arterial elasticity.

For the coronary module, unlike other blood vessels, the coronary vessels reach their peak blood flow during diastole. In order to simulate the special phenomenon of coronary artery, the effect of myocardial contraction needs to be taken into account in the lumped parameter model. The common practice is to add a pressure source synchronized with ventricular pressure into the lumped parameter model. The resistance *R* represents the coronary flow resistance, the resistance *R_m* represents the coronary microcirculation blood flow resistance, and the resistance *R_v* represents the coronary venous flow resistance. Capacitance *C* represents the elasticity of the coronary arteries, and capacitance *C*_*im*_ represents the elasticity of the coronary microcirculation. A voltage source is terminated at the capacitor*C*_*im*_, and the change in value follows the change in left ventricular pressure.

The parameter values of each component in the lumped parameter model are based on the study of coronary artery modeling by Kim et al. (2010). After the structure of the lumped parameter model is determined, the problem of parameter selection should be solved. Firstly, using the data of systolic blood pressure, diastolic blood pressure, heart rate, and cardiac output of normal people, the aorta pressure waveform and cardiac output waveform of normal people were fitted and used as the two optimal target waveforms. In this process, two important points should be noted: (1) the total coronary flow accounted for 4% of the cardiac output, and the left coronary flow and the right coronary flow accounted for 60 and 40% of the total coronary flow, respectively; (2) the blood flow of the coronary artery branch was directly proportional to the 2.7 power of the coronary artery diameter. Secondly, taking the clinically measured aortic pressure and cardiac output as the optimization objectives, the sensitivity analysis was carried out on the parameters of the lumped-parameter model to find the parameters that had great influence on the optimization objectives. Finally, the measured systolic blood pressure, diastolic blood pressure, and heart rate were used to adjust the standard aortic pressure waveform; the root mean square error between the aortic pressure waveform and the simulated waveform, and the mean value of cardiac output were taken as the objective function; and the sensitive parameters in the model were optimized by a simulated annealing algorithm.

After adjusting the parameters according to the normal state, the coronary resistance was multiplied by 0.24 to simulate the maximum hyperemia after adenosine injection when measuring FFR.

This study used a specific interface condition and coupling algorithm to couple the 3D model with the lumped parameter model. The 3D model was calculated by ANSYS-CFX, the set parameter model was calculated based on the FORTRAN subroutine of CFX Junction Box, and the data transfer between the 3D model and the set parameter model utilized the CFX User CEL Function. The lumped parameter model provided a three-dimensional model with flow at the aortic inlet and pressure boundary conditions at each arterial outlet; after the 3D model was calculated, a pressure value was returned to the lumped parameter model at the entrance to the aorta. A flow value was returned to the lumped parameter model at each arterial exit in order for the lumped parameter model to complete the calculation.

Now, the multi-scale model of the 0–3D coupling of the complete coronary hyperemia state is completed. After the calculation, the pressure at the aorta and the distal end of the stenosis was extracted at 3 cm for the calculation of FFR.

### Construction of CABG Model

**FIGURE 3:**
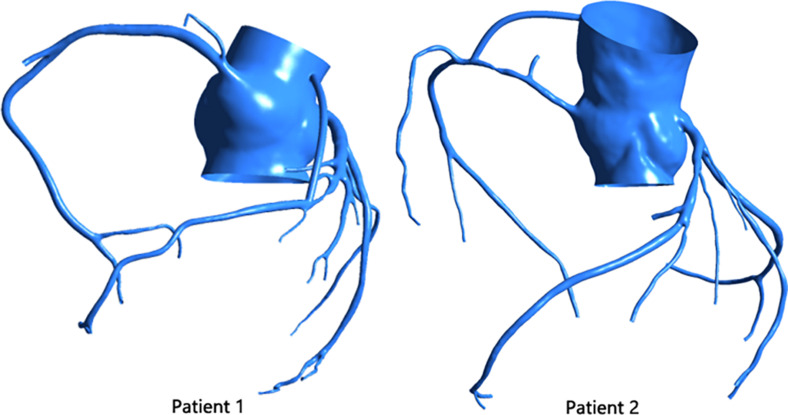
Patient’s CABG model.

The virtual surgery software Freeform was used to graft the stenosis of the two patients. The diameter of the graft was set to 3 mm, and the initial site of the bypass was the aortic root, as shown in Figure 3.

After the 3D model was constructed, the settings of the 0D model part and the 0–3D interface part became the same as the 2.2 part of the method. The difference is that in the CABG model, the coronary artery is in a normal state, so its resistance does not need to be multiplied by 0.24.

## Results

### FFR Non-invasive Calculation Results

**FIGURE 4:**
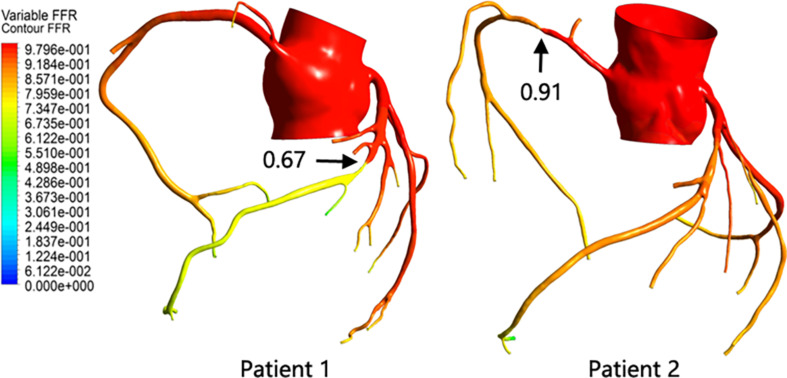
Patient FFR calculation results.

After the calculation was completed, the FFR value of the entire model was extracted. By comparing the FFR diagram of the two models, it can be seen that the color scale of patient 1 at both ends of the stenosis shows a significant difference, indicating that the FFR value has a significant reduction after the stenosis site. The color scales of patient 2 at the front and distal ends of the stenosis site change steadily, and there is no significant difference, indicating that the FFR value does not change significantly after the stenosis site. At the same time, the pressure at 3 cm from the distal end of the stenosis and the pressure in the aorta were extracted, and the ratio was defined as the FFR value of the stenosis. As shown in Figure 4, although the anatomical stenosis rate of both patients 1 and 2 was 70%, their FFR calculation results were quite different. Patient 1 had an FFR of 0.67, and patient 2 had an FFR of 0.91, which are close to their clinical measurements (0.7 and 0.95), indicating that the model reflects the true physiological condition. At the same time, it was also indicated that the stenosis of patient 1 caused downstream ischemia (FFR < 0.75), while the stenosis of patient 2 did not cause downstream ischemia (FFR > 0.8).

### Hemodynamic Environment After CABG

#### Flow of Graft and Coronary Vessels

**FIGURE 5:**
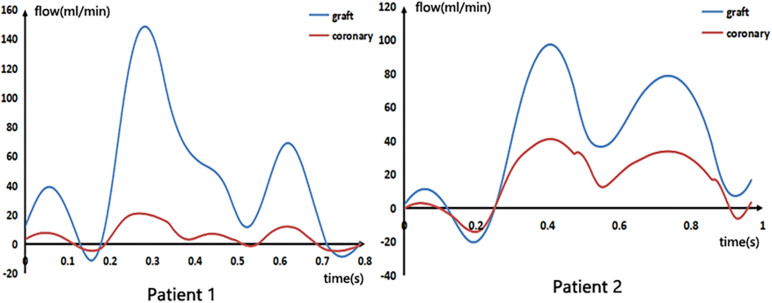
Blood flow waveforms in the graft and stenosis of the patient.

After calculating the CABG model, the blood flow waveforms of the middle segment of the graft and the stenosis coronary artery were extracted, as shown in Figure 5. The mean flow rate, as well as the ratio of stenosis coronary flow to graft flow, are shown in Table 2.

**TABLE 2:** The flow rate of graft and coronary artery.

	**Graft (ml/min)**	**Coronary (ml/min)**	**Ratio**
Patient 1	45.07	5.49	0.12
Patient 2	39.68	16.62	0.42

From the figure and the table, we can see that although the flow rate through the two grafts was not very different, the ratio of the flow rate of the stenosis coronary artery to the graft of patient 1 was 0.12, and the flow ratio of patient 2 was 0.42. This indicates that after the bypass surgery, the proportion of blood supply to the distal coronary artery in patient 1 was large, while the blood supply to the distal coronary artery in patient 2 was still a considerable proportion through the stenosis coronary artery. This reflects the presence of a strong competitive flow in patient 2, which severely reduces blood flow in the graft. Moreover, the negative flow in the waveform of patient 2 was also significantly greater than patient 1, which produces poor hemodynamic results.

#### Results of WSS in the Graft

**FIGURE 6:**
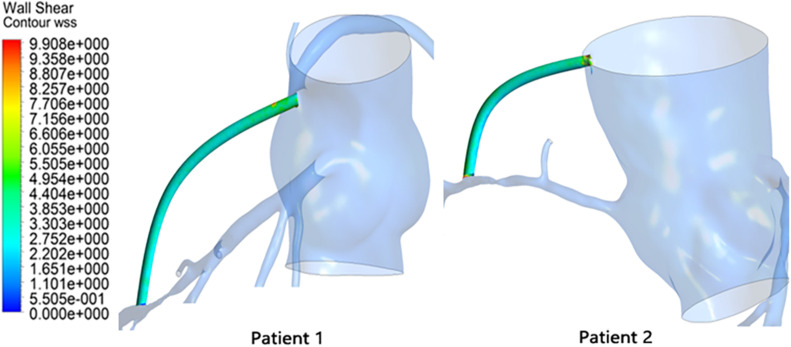
TAWSS results in the graft.

This study extracted time-averaged WSS (TAWSS) results from the grafts, as shown in Figure 6.

The TAWSS in the patient 1 graft was calculated to be 2.09 Pa, and the TAWSS in the patient 2 graft was 2.16 Pa. It can also be seen from the figure that the WSS spatial distributions of the two were similar and were distributed approximately uniformly over the entire graft.

#### Results of Oscillatory Shear Index in the Graft

Oscillatory shear index (OSI) is also a commonly used hemodynamic parameter that characterizes the degree of oscillation of blood flow in a blood vessel, defined as

(4) $$O\,S\,I=0.5\,\left[1-\frac{\left|\int_{0}^{T}\vec{W\,S\,S}\,dt\right|}{\int_{0}^{T}\left|\vec{W\,S\,S}\right|\,dt}\right]$$

**FIGURE 7:**
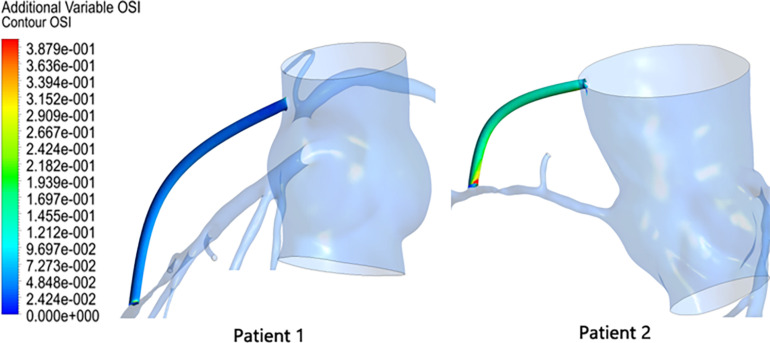
OSI results in the graft.

The OSI results in the graft were extracted as shown in Figure 7. The OSI of patient 1 was calculated to be 0.0375, and the OSI of patient 2 was 0.1264. Patient 2 had an OSI value that was approximately three times that of patient 1. Moreover, according to Figure 7, it can be seen that not only was the mean OSI of patient 2 higher than that of patient 1, but a local high OSI region was also present at the anastomosis.

## Discussion

### Analysis of Factors Affecting FFR

The narrowness of the same stenosis rate (70%) in the image information resulted in different functional results (FFR <0.75 or FFR >0.8). This shows that in addition to the stenosis rate factor, there are other factors that are also affecting stenosis functional outcome. For the two patients in this study, we can see that their stenosis length was not the same; patient 1 had a stenosis length of 10 mm, while patient 2 had a stenosis length of only 4 mm. Within a certain range, a longer stenosis length will result in a larger pressure drop. At the same time, the patients’ heart rate, blood pressure, cardiac output, and other physiological parameters were also different, and these physiological parameters also had an impact on the calculation of FFR. In addition, the location of the stenosis of the two patients was different; the stenosis of patient 1 was in the LAD, and the stenosis of patient 2 was in the RCA. The myocardial area supplied by the LAD is larger than that of the RCA, which means that the distal microvessels of the LAD are more effective than the RCA after adenosine injection, which makes the pressure drop on the LAD larger, which also affects the results of FFR.

### Effect of FFR on Graft

By comparing the blood flow results of the simulated bypass of the two patients, it can be found that the biggest difference between the two was the flow ratio of the stenosis coronary artery to the graft. Patients with high FFR values have a higher ratio of stenosis coronary to graft flow. This means that the coronary artery divides a large part of the blood flow of the graft; that is, the competition flow is strong. However, this did not seem to affect the supply of blood flow to the distal end of the coronary artery. Even when observing the WSS of the graft, it was found that the competitive flow did not cause the WSS to be too low on the graft, nor was the WSS gradient form too large. So, does this mean that we cannot consider the FFR value when we graft? In other words, can we perform a preventive bypass surgery when the FFR shows that the stenosis is not serious?

By continuing to observe the results of OSI in the grafts, we found that this is not the case. By performing a bypass surgery on a high FFR stenosis, the OSI value in the graft is significantly higher. In this study, the OSI value in the patient 2 graft was three times that of patient 1. This will result in poor long-term permeability of the high FFR-narrow graft, and this inference is consistent with the clinical observations mentioned in the section “Introduction” of this article. At the same time, we can observe that the OSI in the graft is not evenly distributed but has a very high OSI region near its anastomosis. This will lead to the formation of intimal hyperplasia at the anastomosis, which will block the blood supply downstream of the coronary artery. Therefore, from a long-term perspective, prophylactic grafting when the FFR shows that the stenosis is not severe does not improve the supply of blood flow but also worsens the original blood supply.

To sum up, the most important role of FFR technology in CABG is to guide bypass surgery and decide whether to bypass a stenosis site. A purely anatomic diagnosis would result in a graft to a site of moderate stenosis, which would result in the failure of the graft. FFR, a functional diagnostic method, can avoid these failed bypass operations and improve the graft patency.

### Limitation

In this paper, the relationship between FFR and graft hemodynamics was investigated using the data of two patients. This amount of data are insufficient to draw conclusions, and it was difficult to draw the cutoff value of FFR to determine whether a stenosis was suitable for bypass. In future studies, more cases should be introduced to verify the results, and a reasonable FFR cutoff value guiding coronary bypass surgery can also be explored.

## Conclusion

When bypassing a stenosis with high FFR value, its adverse hemodynamic effects were mainly reflected in OSI. It produced a high OSI region in the graft, especially at the anastomosis. In the long term, this will cause anastomotic blockage and graft failure. FFR, a functional diagnostic method, can be introduced to avoid failed bypass surgery. Therefore, a quick evaluation of the CABG using FFR may be considered prior to bypass surgery, which will help improve the graft patency.

## Data Availability Statement

All datasets generated for this study are included in the article/supplementary material.

## Ethics Statement

This study passed the inspection by the medical ethics committee of Peking University people’s hospital. All participants gave signed informed consent.

## Author Contributions

BL was responsible for model calculation and manuscript revising. BM was responsible for modeling, calculation, data analysis, and article writing. YF assisted in data analysis and article writing. JL assisted in the establishment of the three-dimensional model. ZZ was responsible for providing experimental data. MD assisted in the article writing. YL was responsible for supervision. All authors contributed to the article and approved the submitted version.

## Conflict of Interest

The authors declare that the research was conducted in the absence of any commercial or financial relationships that could be construed as a potential conflict of interest.
